# Medical overuse and quaternary prevention in primary care – A qualitative study with general practitioners

**DOI:** 10.1186/s12875-017-0667-4

**Published:** 2017-12-08

**Authors:** Kathrin Alber, Thomas Kuehlein, Angela Schedlbauer, Susann Schaffer

**Affiliations:** Institute of General Practice, Friedrich-Alexander-University Erlangen-Nuernberg (FAU), Universitaetsstr. 29, 91054 Erlangen, Germany

**Keywords:** Medical overuse, Over investigation, Overtreatment, Quaternary prevention

## Abstract

**Background:**

Medical overuse is a topic of growing interest in health care systems and especially in primary care. It comprises both over investigation and overtreatment. Quaternary prevention strategies aim at protecting patients from unnecessary or harmful medicine. The objective of this study was to gain a deeper understanding of relevant aspects of medical overuse in primary care from the perspective of German general practitioners (GPs). We focused on the scope, consequences and drivers of medical overuse and strategies to reduce it (=quaternary prevention).

**Methods:**

We used the qualitative Grounded Theory approach. Theoretical sampling was carried out to recruit GPs in Bavaria, Germany. We accessed the field of research through GPs with academic affiliation, recommendations by interview partners and personal contacts. They differed in terms of primary care experience, gender, region, work experience abroad, academic affiliation, type of specialist training, practice organisation and position. Qualitative in-depth face-to-face interviews with a semi-structured interview guide were conducted (*n* = 13). The interviews were audiotaped and transcribed verbatim. Data analysis was carried out using open and axial coding.

**Results:**

GPs defined medical overuse as unnecessary investigations and treatment that lack patient benefit or bear the potential to cause harm. They observed that medical overuse takes place in all three German reimbursement categories: statutory health insurance, private insurance and individual health services (direct payment). GPs criticised the poor acceptance of gate-keeping in German primary care. They referred to a low-threshold referral policy and direct patient access to outpatient secondary care, leading to specialist treatment without clear medical indication. The GPs described various direct drivers of medical overuse within their direct area of influence. They also emphasised indirect drivers related to system or societal processes. The proposed strategies for reducing medical overuse included a well-founded wait-and-see approach, medical education, a trustful doctor-patient relationship, the improvement of primary/health care structures and the involvement of patients and society.

**Conclusions:**

GPs are frequently located at the starting point of the diagnostic and treatment process. They have the potential to play a vital role in quaternary prevention. This requires a debate going beyond the medical profession and involving society as a whole.

**Electronic supplementary material:**

The online version of this article (10.1186/s12875-017-0667-4) contains supplementary material, which is available to authorized users.

## Background

Medical overuse has become a problem of increasing importance to patients and health care systems. A variety of interrelated or even competing concepts of medical overuse exists and a consensus on a detailed terminology is not yet achieved [1]. Morgan et al. summarise definitions of medical overuse including overdiagnosis, diagnosis of abnormalities not related to disease, unnecessary medical evaluation, overtesting, overtreatment, wrong practice or unwanted care – processes which could be provider- or patient driven [2]. There is a growing awareness among clinicians and health care scientists, that medical overuse comprises unnecessary health care lacking benefit for patients [3] or putting them at risk of harm outweighing a potential benefit [4]. Moreover, unnecessary medicine adds to rising health care expenditures [5] and a misallocation of scarce resources [6]. Asymptomatic individuals are at risk of being labelled as patients, causing anxiety and affecting their quality of life [7].

Moreover, in secondary prevention, risk factors are increasingly treated as diseases [8]. There is a tendency to screen asymptomatic populations at low risk and to label pre-diseases as manifest diseases [1]. Serum cholesterol levels are a good example of threshold lowering by shifting the boundary between health and disease [9].

**Fig. 1:**
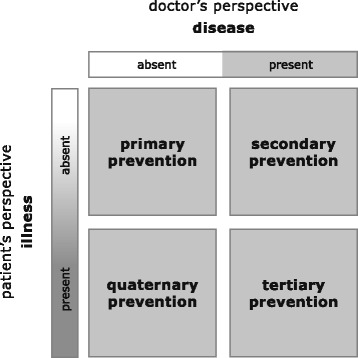
The concept of quaternary prevention. Source: [11] Kuehlein T, Sghedoni D, Visentin G, Gérvas J, Jamoulle M. Quaternary prevention: a task of the general practitioner. PrimaryCare. 2010;10:350–4, and [12] Jamoulle M. Quaternary prevention, an answer of family doctors to overmedicalization. Int J Health Policy Manag. 2015;4:61–4

A growing number of research and health care campaigns show the worldwide importance of this topic. “Choosing wisely”, an initiative founded in 2015 by the American Board of Internal Medicine, addresses unnecessary medical procedures via top five recommendation lists in cooperation with a growing number of specialty societies [10]. In primary care, the “quaternary prevention concept” [11] was introduced (see Fig. 1 **)** in order to protect individuals from unnecessary investigations and treatment. Quaternary prevention is a “new term for an old concept: first, do not harm” [12]. It refers to actions “taken to identify [a] patient at risk of overmedicalisation [= in the sense of medical overuse, author’s note], to protect him from new medical invasion, and to suggest to him interventions, which are ethically acceptable” [13].

The complex influencing factors of medical overuse and the possibilities to reduce it have to be interpreted in a system-specific context. The German health insurance system comprises several coverage schemes for out-patient and in-patient care: First, the statutory health insurance (about 86% of residents), financed by compulsory contributions as a percentage of income (up to an income ceiling) including employed persons and their non-earning dependents (free of charge). Second, private health insurance (about 11% of the population) for persons above the income threshold for compulsory insurance (opt out), self-employed persons and civil servants.^1^ Patients have free access to primary care, out-patient secondary care and in-patient care (with referral), including diagnostics, treatment and preventive services. There is no mandatory enrolment in a specific primary care model. There has been an ongoing discussion on a compulsory gate-keeping function of GPs, but to date, this has not been implemented. Health care spending also includes out-of-pocket spending and co-payment (pharmaceuticals, aids, in-patient treatment). In out-patient care, so-called individual health services are offered. These extra services are not included in the statutory health insurance coverage and are reimbursed as direct payment (out-of-pocket) to the provider [14].

The primary care perspective plays an important role in tackling the challenge of unnecessary medicine. General practitioners (GPs) face an unselected patient population with a low prevalence of manifest disease. They are confronted with a higher level of uncertainty regarding the correct and final diagnosis [11]. Preventing patients from broad diagnostic testing for unspecific symptoms [15] is an essential task in this setting: Once a cascade of unnecessary medical interventions has been started, it might almost become impossible to stop it [16].

Although a broader research agenda on medical overuse has been suggested [2], there has been little focus on primary health care. This is why our study aims at a deeper understanding of the definitions of medical overuse, its scope, consequences and its drivers, as well as quaternary prevention strategies from the GPs’ point of view. We were especially interested in the aspects of medical overuse within the GPs’ direct area of influence.

## Methods

### Study design

**Fig. 2:**
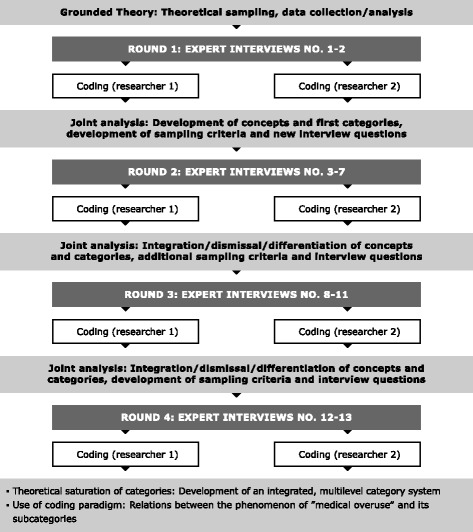
Interrelated process of theoretical sampling, data collection and analysis

According to the explorative nature of our research question, we chose a qualitative study design and used the Grounded Theory approach by Strauss and Corbin [17]. In Grounded Theory, sampling, data collection and analysis are defined as an interrelated and iterative process [18]. Figure 2 shows the interrelated process of sampling, data collection and analysis.

### Sampling and recruitment

Theoretical sampling [17] was performed in order to achieve a maximum variation of concepts and categories which describe the phenomenon of medical overuse. In total, 13 GPs who work within the German health care system were included in the study. The development of sampling criteria was a combined deductive and inductive process. We focused on a sampling based on relevant data to identify, substantiate, dismiss or broaden concepts and categories. Due to an initially easier access to the field of research, we first recruited GPs who had an affiliation to the Institute of General Practice, Friedrich-Alexander-University Erlangen-Nuernberg (FAU), taking the sampling criteria age/work experience into account. Through personal contacts and recommendations by interviewed GPs we found additional interview partners with similar or different opinions or behaviour related to the phenomenon of medical overuse. Throughout the course of the study, the sampling was done stepwise and empirically-driven as theoretical sensitivity increased [18]. Finally, GPs differed with regard to the following sampling criteria: years of primary care experience, gender, region (urban/rural), work experience abroad (in our study: none, UK, Ireland, Denmark, Guatemala), academic affiliation to an institute of general practice, type of specialist training, practice organisation and position (group/single practice, practice owner/employee). Sampling was continued until theoretical saturation [17] of information was achieved. Participants were approached face-to-face and via email. There were no drop outs. Prior to the interview, the involved GPs received a fact sheet including a description of the research goals, the interviewer, the research team and data protection.

### Data collection

For data collection, the method of in-depth expert interviews based on a semi-structured guide with mostly open-ended questions was chosen. The interview guide was pilot tested. At the beginning, we only had a few open interview questions, always depending on the interview partner’s fluency. The guideline was extended and adjusted (more back-up questions and prompts) with growing insight into the research topic throughout the course of the inquiry [17]. Main interview topics (see Additional file 1) were a) relevance and definitions of medical overuse, b) examples of unnecessary investigations and treatment in own daily work routine and/or observed in other colleagues, c) drivers and consequences of medical overuse, d) strategies to prevent patients from medical overuse in general and/or applied by the interviewed GP (quaternary prevention). In order to reduce negative effects of social desirability, the interview questions started with the GPs’ observations and opinions in general or also their perception of other GPs’ work routines. In a second step, they were encouraged to reflect on their own potential contribution to medical overuse. All interviews were conducted by the same interviewer (KA), face-to-face at the GPs’ workplace. They had an average duration of 64 min, were tape-recorded and transcribed verbatim in German. The translation process of the quotes used in this manuscript was supervised by one of the authors (AS) with long-standing work experience within the NHS primary care services and UK health care sciences.

### Data analysis

Two independent researchers (KA and TK) coded the transcripts sequentially. After interview 2, 7, 11 and 13, the codes were compared with each other and either consolidated or further specified adding new interview questions (investigator triangulation). Analysis was supported using RQDA software [19]. We applied the following interrelated coding approaches:

First, open coding [17] lead to data-based concepts which were grouped in categories on a higher level. We examined our data for concepts, categories and its properties and dimensions. We used the method of constant comparison to substantiate those categories and to develop new interview questions. Flip-flop techniques (for example in terms of the rationale of medical overuse to be located between necessary economic survival and profit maximisation) were applied. A special focus lay on red flags (one GP held the belief that medical overuse only takes place in secondary care, not in primary care, for example). The extent to which we added new interview questions to differentiate and specify newly found properties and dimensions again was defined according to its importance to help explain the phenomenon of medical overuse.

Second, the coding paradigm used in axial coding helped to relate the data-based categories to subcategories (causal conditions, context and intervening conditions, strategies and consequences) to get a deeper understanding of the phenomenon of medical overuse. Due to the immense amount of categories we found, we were not able to give a full, comprehensive figure (based on the coding paradigm), but included figures and tables to the individual results paragraphs instead. Throughout the course of the analysis, the method of constant comparison [18] allowed to challenge existing concepts and relations between categories with new data. It helped to avoid bias in the combined inductive-deductive analytical process and improved the precision, consistency and congruence of emerging concepts and its relations [18].

We continued this iterative process until theoretical saturation [17] of information was achieved and answers kept repeating (see timeline in Additional file 2 as an example for this iterative process and theoretical saturation of information). Memo writing in terms of categories, properties, hypotheses and newly evolved interview questions was applied [18]. It led to an integrated and multi-level classification system of concepts and categories related to the phenomenon of medical overuse.

In order to conduct a full Grounded Theory study, selective coding and theory building as a third coding step could be applied. Strauss and Corbin [17, see axial coding chapter] explain that in case of thematic analysis or development of concepts, open and axial coding might be sufficient. As there has been little research background on the topic of medical overuse in German primary care, we think it would be too early to present a theory of this phenomenon yet. Our focus lay on a comprehensive description of its scope and context, relevant drivers, conditions, consequences and strategies to avoid it.

## Results

### 1. Overview

#### 1.1 Main results

The main results of our inquiry are related to GPs’ definitions of medical overuse, its scope and consequences, including examples of over investigation and overtreatment and also the country-specific aspect of referrals to secondary out-patient care. We then focus on direct and indirect drivers of medical overuse (related to a direct or indirect area of influence from a GP’s perspective), paying attention to vulnerable groups. Finally, various strategies for reducing medical overuse from the interviewed GPs’ point of view are described.

#### 1.2 Study population

**Fig. 3:**
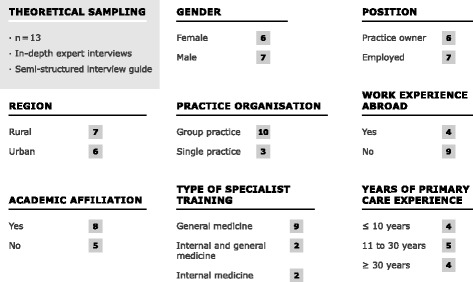
Study population

Background information and demographics of the study population (see further information in the methods section) are shown in Fig. 3.

## 2. GPs’ definitions of medical overuse

Although there was a broad variation in the GPs’ perception of medical overuse, all had experienced it in their working environment. They generally defined medical overuse as unnecessary medicine that lacks benefit for patients or could even harm them in terms of morbidity, health-related quality of life or mortality. They distinguished between a number of negative consequences: First, they observed a spiral of diagnostic testing including short re-visit intervals without patient benefit or treatment consequences. Second, in their opinion, unnecessary testing increased the risk of finally irrelevant incidental findings, which nonetheless required further diagnostic workup. Third, they saw the risk of rising health care expenditures. Fourth, GPs were concerned that medical overuse promotes a misallocation of scarce resources that leads to undertreatment in other health care areas.

*Quotation: “There is always the danger of harming my patient. For example with certain prevention examinations such as screening for mamma carcinoma which is up for discussion at the moment.*” *(General Practitioner, A11).*

*Quotation: “People are suddenly confronted with diagnoses and diseases, where nobody knows, whether it is a disease or not. A debate which already started in screening. You pay a high price trying to examine everything and everyone.” (General Practitioner, A06).*

*Quotation: “(…) through this approach (author’s note: extended laboratory diagnostics) you sometimes find borderline values, which require subsequent explanations.*” *(General Practitioner, A01).*

*Quotation: “Would it have consequences which change our further treatment? Or do we get results which do not change our actions? Then we must ask ourselves: do we need this test at all?” (General practitioner, A09).*

Their definition was either acquired by evidence from study findings (in the sense of formal, published peer reviewed evidence) or, in a more subjective approach, by their individual expertise from personal work experience. Some of the interview partners emphasised the challenge of clear inclusion and exclusion criteria in the search for a sound definition of medical overuse and quaternary prevention strategies.

*Quotation: “The reference base would be, as almost always in our profession: Does it make a difference? Sometimes, doing more leads to a better outcome for patients. And sometimes it does not.” (General practitioner, A06).*

*Quotation: “Through this interview, I became aware again of the difficulties to define medical overuse. It is complex. (…) There will always be borderline cases with which it is difficult to decide between action and wait-and-see.” (General practitioner, A09).*

Regarding the debate on medical overuse, some of the interviewed GPs argued for a shift of emphasis to other health care problems such as under-supply in terms of health care financing, undertreatment, doctor shortage and bureaucracy in the health care system. One GP feared that the overuse debate could pave the way for rationing health care resources.

*Quotation: “There are debates that the same number of cars could be produced in two factories instead of four factories. Nobody poses the question, whether we actually have a financing problem or not. Hospital beds are used to capacity, nurses complain about overwork, physicians grumble about too much bureaucracy.” (General practitioner, A09).*

## 3. The scope of medical overuse in primary care

### 3.1 Examples of medical overuse

The GPs were of the opinion that medical overuse in primary care is a topic broad in scope and relates to both over investigation and overtreatment. They observed that medical overuse takes place in all three reimbursement categories within the German health care system: Diagnostic and treatment procedures covered by the German statutory health insurance; health care for patients with a private health insurance that reimburses about twice as much as the statutory health scheme and accepts a broader range of investigations and treatment; and finally diagnostic and treatment procedures in the context of the so-called “individual health services” which are covered by direct payment. Table 1 shows examples of over investigation and overtreatment from the perspective of the interviewed GPs. The boundaries between the three main reimbursement categories are overlapping – the stated examples occur in all the three health schemes.

**Table 1:** The scope of medical overuse in primary care

Statutory health insurance ↔ Individual health services ↔ Private health insurance
Over investigation
Laboratory diagnostics
Holter-ECG/Long-term blood pressure control
Ultrasound (thyroid, abdomen)
General check-up in asymptomatic patients
PSA screening in asymptomatic patients
“Extended prevention investigation” in asymptomatic patients: Ultrasound, ECG, ergometry, spirometry, laboratory diagnostics
GP-recommended self-diagnostic devices (blood glucose/blood pressure)
Overtreatment
Polymedication in general (often discrepancies after hospital discharge)
Physical therapy covered by health insurance for new unspecific back pain
Broad-spectrum antibiotics for uncomplicated community acquired pneumonia (CAP)
Injections for back pain relief
Antibiotics for viral infections
Long-term prescription of benzodiazepines
Opioid therapy in chronic non-cancerous pain
Vitamine replacement therapy
Referrals to out-patient secondary care: GP-, specialist- or patient-driven
Specialist visits for basic medical problems: uncomplicated hypertonia, diabetes, new unspecific back pain
Referrals to MR Imaging for new unspecific back pain

A specific emphasis was placed on prevention and screening services such as medical check-ups and PSA-testing for screening of prostate cancer. In Germany, all patients with statutory health insurance coverage can make use of a biennial preventive check-up from the age of 35 (general medical examination). Some of the GPs criticised a lack of evidence regarding patient-centred outcomes for these examinations and a missing standardisation that leads to a varying check-up quality. Moreover, they frequently experienced an adverse selection of asymptomatic patients with a health-conscious, low-risk lifestyle, whereas patient groups with higher risks were underrepresented. Some also disapproved of PSA screening in asymptomatic patients, referring to clear evidence against screening.

*Quotation: “As I said, a patient without previous medical history, without symptoms. In this case, I have never auscultated a lung and thought: “Thank god I listened to that lung.” I mean, what do you expect from a healthy patient when you auscultate the lung? A healthy lung.” (General Practitioner, A01).*

### 3.2 Low priority of primary care and gate keeping in Germany – Medically unnecessary referrals?

In Germany, primary care has not been given much gate-keeping responsibility as this is the case in other health care systems. Secondary care is carried out not only in the in-patient, but also in the out-patient sector. Moreover, German patients have direct access to secondary out-patient care without seeing a GP first. The interviewed GPs reported on frequent presentation of patients to secondary care specialists (especially in the out-patient sector) either based on patient self-referral or GP referral without a clear medical indication as an important dimension of medical overuse (see Table 1 for examples). The interviewed GPs were pointing out various drivers for this aspect of unnecessary medicine. Direct factors related to GPs themselves were: The fear of making mistakes, little capacity of coping with residual uncertainty and finally the possibility of placing the responsibility for strenuous patients to other colleagues.

*Quotation: “I’ve been grateful for my whole life that there are secondary care specialists. I have never wanted to be the last link in the chain, the one to decide about the final diagnosis. That’s why I did not go into anaesthesiology, although I really liked it. Because I thought, there you are the last to decide, everything is up to me. That is not my personality. There is this insecurity, yes.” (General Practitioner, A13).*

The interview partners also pointed to reasons of medically unnecessary referrals which could not be influenced by the GPs themselves (indirect factors): So-called “circle referrals” made by secondary care specialists within the office-based ambulatory sector which undermine the role of the GP both as a family doctor and a gate-keeper; narrow follow-up intervals (every three months) in the out-patient secondary care sector; patients with a considerable need for reassurance; patients considering secondary care specialists as better qualified to deal with medical problems in general; and finally insufficient time for the individual patient due to an inadequate capitation fee per patient.

*Quotation: “I have experienced so-called “circle referrals” [author’s note: referrals between secondary care specialists in the out-patient sector]. There exist some kinds of networks. In this context, there is medical overuse based on a missing flow of information. And in this case, there is no overview over the patient’s entire diagnostics, treatment and risks related to it, especially when medical problems occur outside of the specialist’s focus.” (General Practitioner, A11).*

## 4. Drivers of medical overuse from a primary care perspective

**Fig. 4:**
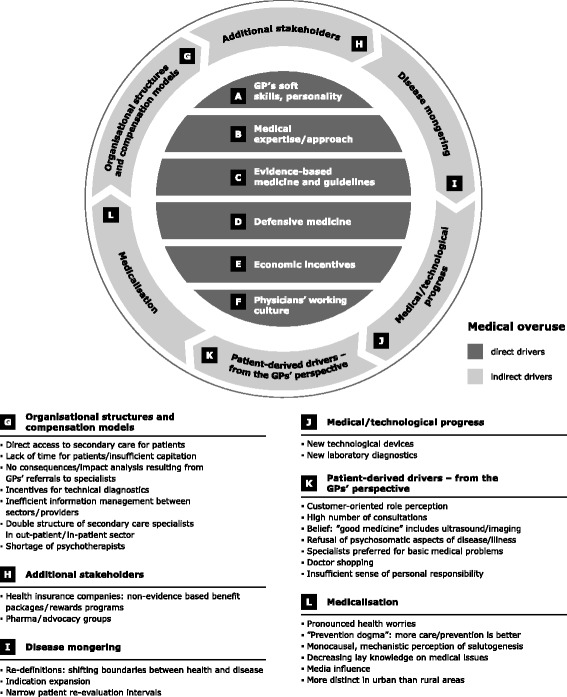
Drivers of medical overuse in primary care

The participating GPs were asked about their beliefs and observations regarding drivers of medical overuse. They reported on manifold drivers of unnecessary medicine (see Fig. 4), which could be related – from the GPs’ perspective – to a multilayer continuum of direct or indirect areas of influence (in dependence on Morgan’s classification of direct and indirect drivers [2]). The study focus lay on aspects being located within the GPs' direct area of influence. These direct drivers are described in detail below, followed by a brief description of indirect drivers.

### 4.1 Direct drivers

#### A. GPs’ soft skills and personality

Some of the interviewed GPs believed that a lack of empathy or communicative skills and a dysfunctional doctor-patient relationship that loses sight of the individual patient’s needs could promote medical overuse. Additionally, many of the GPs were of the opinion that a missing ability to accept well-founded “wait and see” could lead to unnecessary medicine. In this context, they considered the following factors to be influential: A refusal of GPs to take on responsibility for the medical process, an insecure personality structure with low professional self-confidence and a low level of ambiguity tolerance in the sense of an inability of coping with residual uncertainty.

*Quotation: “You have to be self-confident. Actually, being self-confident in taking actions is not the point. You have to be self-confident in not doing something.” (General Practitioner, A12).*

#### B. Medical expertise/approach

Some GPs stated that they were prone to medical overuse in their first years on the job because of their uncertainty due to a lack of medical work experience. Many of the GPs pointed out that a low quality of medical education and training could result in diagnostic uncertainty and finally promote unnecessary medicine. A growing tendency towards (sub-)specialisation and a low priority of history taking and physical examination were also seen as major drivers for the maximisation of technical diagnostics. GPs criticised that the absence of a holistic patient assessment which includes overall morbidity, health-related quality of life and psychosocial factors promotes medical overuse.

*Quotation: “The greatest challenge will be to put more emphasis on history taking and physical examination again. If a young physician learns how to observe clinical symptoms and adequately acts on it, he will feel secure. This is the prerequisite to avoid further unnecessary investigations.” (General Practitioner, A02).*

#### C. Evidence-based medicine and guidelines

The interviewed GPs’ approach to medical decision-making varied. Some held the opinion that medical decision-making should be based on evidence as a way to avoid medical overuse, allowing for deviations of guidelines in individual cases.

*Quotation: “In an ideal world, GPs would cooperate. We would establish evidence-based guidelines for primary care, we would work together, we would work in group practices. Organise our practices according to the best guidelines available and care about us and for our patients.” (General Practitioner, A04).*

Some answers showed a reasonable knowledge and approval of evidence-based medicine, but also revealed a lack in adherence. Either GPs did not feel responsible for managing an overall evidence-based care for their patients or they even appreciated medical overuse in inpatient care as a welcome diagnostic work-up and baseline for the subsequent outpatient care.

*Quotation: “When a patient gets discharged from the hospital, I sometimes have the feeling that they did anything medically possible. But from the out-patient care perspective, I also appreciate that. (…) At the very moment a patient starts showing symptoms, I know, the patient has been in in-patient treatment recently. And then I am glad that I can refer to something. (…) And you could describe that as medical overuse to some extent. Because we are talking about tests which were not totally urgent or rather luxurious given the specific symptoms at that time. But it can be really helpful to have this reference point.” (General Practitioner, A11).*

In some of the GPs, a lack of acceptance or willingness to reflect the principles of evidence-based medicine could be observed. Other GPs even spoke of a blind spot in this context. Contrary to current guidelines, some of the interviewed GPs overestimated the benefit of certain medical procedures and contributed to medical overuse. This was especially noticeable when talking of the benefits of PSA testing or general medical check-ups in asymptomatic persons. There were several reasons why physicians overruled current guidelines and insisted on their freedom of therapy and the individuality of each patient: First, the misinterpretation of statistical evidence; second, an “action” dogma of doing anything that is possible for the individual patient; third, a belief that screening and prevention is always something good; fourth, an over-reliance on (causal) treatment effects; and fifth, a low visibility of negative consequences of medical overuse.

*Quotation: “In my opinion, it would make sense to allow general practitioners to do anything possible when carrying out a general medical examination. In terms of prevention. Intima-media thickness, for example. Ultrasound, too. Once in a while, you find renal tumours there. You only find them accidentally when looking for something else. Otherwise you would miss them. I think any patient you identify is a life saved.” (General Practitioner, A08).*

*Quotation: “In my opinion, those things which help, those who cure the patient are right. And when there is a patient who tells me that my vitamin therapy helped him, I don’t adhere to studies saying it is useless.” (General practitioner, A13).*

In general, all groups described problems arising with the development of evidence-based guidelines such as vested interests, limited applicability to the individual patient and a tendency to promote action rather than inaction. A timeline of interviews combined with a mapping of answers and categories to the topic of “Evidence-based medicine and guidelines” is provided in Additional file 2. It also shows theoretical saturation of information within the study population.

#### D. Defensive medicine

Some interview partners pointed to the fear of liability as an important reason for unnecessary diagnostic tests. Although none of them reported on personal experience of legal actions against them, this aspect played an important role from the GPs’ point of view.

*Quotation: “Another reason is that diagnostic tests are also carried out due to a fear of liability. To protect oneself from claims of compensation.” (General Practitioner, A03).*

#### E. Economic incentives

All interview participants referred to economic drivers of medical overuse. Some GPs reported that they themselves or colleagues carried out unnecessary tests and treatments for reimbursement reasons. The rationales given were located between necessary economic survival and profit maximisation. Some GPs described the existence of provider-induced demand that would lead to non-evidence-based care.

*Quotation: “There is little transparency among general practitioners in terms of income. It was often presented as a basic economic necessity to increase turnover with these services [non-evidence based individual health services with direct payment; annotation by the author]. Personally, I am not dependent on that.” (General Practitioner, A01).*

#### F. Physicians’ working culture

Some of the GPs stated that there is little individual scope for structural change. Eminence-based medicine, hierarchical structures and a lack in debate culture were also seen as drivers for medicine without patient benefit. In general, a paradigm shift from action to inaction in well-founded cases was assessed as essential but hard to achieve.

*Quotation: “In the Anglo-Saxon countries they practice evidence-based medicine, we Germans carry out eminence-based medicine. It has been like this for a long time, when chief physicians were gods and everybody had to believe them.” (General Practitioner, A04).*

### 4.2 Indirect drivers (G-L)

The interviewed GPs also referred to drivers which were not located within their direct area of influence (see Fig. 4). From the GPs’ perspective, these indirect aspects included (G) organisational structures and compensational models which could promote medical overuse; (H) additional stakeholders such as health insurance companies or pharmaceutical industry that primarily act on economic prerequisites instead of avoiding unnecessary medicine; (I) disease mongering [1], a process that shifts boundaries between health and disease; (J) the medical and technological progress expanding diagnostic and treatment options; (K) patient-derived drivers, relating to patient demands which arise from a multidimensional setting of personal belief systems, dysfunctional incentives and sociocultural developments; and (L) a general process of medicalisation of the society as a whole, affecting all stakeholders such as the public, politicians, doctors, patients, health insurances, pharma industry or other advocacy groups.

### 5. Vulnerable groups

The interviewed GPs identified various vulnerable groups who in their view are especially at risk of medical overuse: First, the so-called “worried well” [11], individuals with no or minor symptoms who frequently see a doctor; second, patients in a palliative situation who receive maximum investigations and treatment instead of a quality of life-focused symptom-oriented approach; third, patients with psychosomatic diagnoses who focus on somatic health care instead of psychotherapy; and fourth, old and multimorbid patients who are at risk of polypharmacotherapy potentially leading to severe side effects.

*Quotation: “Patients in palliative situations are at risk of medical overuse. For them, it makes no sense to do anything that is medically possible.” (General Practitioner, A05).*

*Quotation: “In primary care, we have many patients with diseases that are no diseases or only minor disturbances.” (General Practitioner, A08).*

### 6. Quaternary prevention: Strategies for reducing medical overuse

Derived from their perspective on the scope and drivers of medical overuse, the participating GPs came forward with a variety of recommendations to reduce medical overuse in primary care (see Table 2). These strategies could be related – from the GPs’ perspective – to a multilayer continuum of direct, intermediate and indirect levels of influence.

**Table 2:** Quaternary prevention from a primary care perspective

Quaternary prevention strategies: Levels of influence		
A. DIRECT	B. INTERMEDIATE	C. INDIRECT
A1. Establishing a trustful doctor-patient-relationship	B1. Promotion of a primary care-centred health care model	C1. Improving health care structures
Focus on a long-term relationship between doctor and patient	Improvement of evidence-based primary care	Restriction of non-evidence-based individual health services (direct payment)
Shared-decision-making	GP as guide and coordinator	Population-based health care approach (instead of an extended high risk approach)
Improving soft skills (communication, empathy)	Distinction between primary and secondary care	Change in reimbursement paradigms: less incentives for technical diagnostics
Holistic patient assessment (including the patient’s social background)	Better integration of primary care into medical school curriculum	
A2. Reducing diagnostic uncertainty	B2. Patient education	C2. Discussion in society as a whole
High quality, evidence-based medical education and training	Information on evidence for recommended or requested services	Identification of relevant stakeholders
Supervision for young GPs	Information on advantages of a wait-and-see-approach instead of immediate maximum diagnostics	Process of setting priorities in health care
Stepwise diagnostics: Focus on anamnesis and physical examination	Information on importance of health-conscious behaviour/personal responsibility	
Well-founded “wait and see”	Price/cost transparency	

In terms of a *direct* level of influence (A), they outlined manifold quaternary prevention strategies: Establishing a long-standing, trustful doctor-patient-relationship, based on shared-decision-making, an emphasis on communication and empathy for the patient and a holistic patient assessment could help downsizing unnecessary medical procedures. A focus on reducing diagnostic uncertainty through high-quality education and training, supervision especially for young GPs and carrying out stepwise diagnostics with a focus on anamnesis and physical examination and a well-founded “wait and see”-strategy instead of an early-stage shotgun testing were also seen as essential quaternary prevention strategies. On an *intermediate* level of influence (B), the promotion (through GP associations) of a more primary care-centred health care model and patient education were considered as important. On an *indirect* level of influence (C), the importance of improving health care structures and discussing health care priorities in society as a whole was emphasised.

The interviewed GPs also stressed their limited influence and the decisive power of various other stakeholders (health policy, statutory health insurance, media or the pharmaceutical industry).

## Discussion

### 1. Principal findings

According to our main research questions, the interviewed GPs gave numerous definitions of medical overuse, comprising over investigation and overtreatment. They defined medical overuse as unnecessary investigations and treatment which lack benefit for patients or could even harm them in terms of morbidity, health-related quality of life or mortality. The interview partners were of the opinion that medical overuse occurs in all three reimbursement categories within the German health care system: the statutory health insurance, private health coverage and payments covered by the patients individually (“individual health services”). Especially PSA screening and medical check-ups were seen as controversial. GPs criticised the little acceptance of gate-keeping in German primary care and a low-threshold of referral, in particular the direct accessibility of secondary care. In their opinion, these special characteristics of the German health care system contribute to medical overuse, leading to (a) GP-driven referrals without clear medical indication, (b) “circle referrals” within the out-patient secondary care sector or (c) patient-driven direct visits to out-patient secondary care specialists.

They pointed to various drivers of medical overuse being associated either directly or indirectly with their areas of influence. Direct drivers promoting unnecessary medicine comprised a lack of consultation skills, such as empathy and an ability to communicate, insecure personality traits, a lack of medical experience, little acceptance of guidelines, a high degree of defensive medicine, economic incentives and the physicians’ working environment with its lack of debating culture and inherent hierarchies in general. They also referred to indirect drivers of medical overuse, such as dysfunctional incentives in terms of organisational structures and compensation models, interests of additional stakeholders (health care companies or the pharmaceutical industry), disease mongering [1], medicalisation, patient demands and the medical and technological progress.

Four vulnerable patient groups who especially are at risk of medical overuse were identified: The so-called “worried well” [11] seeing a doctor frequently, patients in palliative care situations, patients with psychosomatic diseases and old, multimorbid patients who are at risk of polypharmacotherapy.

The interviewed GPs proposed a number of strategies to reduce medical overuse in primary care (quaternary prevention). Strategies which could be directly applied by GPs themselves (direct level of influence) comprised an improvement of the doctor-patient-relationship through shared-decision-making, communication and a holistic patient assessment. These strategies also included the reduction of diagnostic uncertainty through high-quality training for GPs, a well-founded wait-and-see approach and stepwise diagnostics with a focus on history taking and physical examination, for example. On an intermediate level of influence, strategies emphasised the development of a primary care-centred health model and the importance of patient education. On an indirect level of influence, adjusting health care structures and discussing health care priorities were seen as essential.

### 2. Findings in relation to other studies

The definitions, scope, drivers and also the outlined quaternary prevention strategies we found, coincided with international findings to a large extent. However, there were country-specific German particularities, especially the extensive use of ultrasound in general practices, the amount of referrals to specialists due to non-medical reasons or the uncontrolled direct access to secondary care for patients themselves.

The GPs’ heterogeneous evaluation of check-up tests in asymptomatic individuals corresponded to findings from other countries such as Ireland [20]. In terms of reasons for medically unnecessary referrals made by GPs, our results coincided with Wammes et al. who also found a fear of making mistakes, lack of time and demanding patients to be major drivers [21].

The importance of drivers such as malpractice claims, financial incentives for diagnostic testing and too little time for patients corresponded to the findings of a representative study with US primary care physicians [22]. However, the impact of fear of malpractice claims on medical overuse is controversially discussed in literature. Levinson et al. reported on the importance of a well-functioning doctor-patient-relationship for the decrease of potential lawsuits [23].

Other findings showed the concerns the interviewed GPs raised over complex and potentially biased guidelines. A review of NHS clinical practice guidelines from 2010 and 2011 analysed that publications which formed the evidence basis for these guidelines were in 62% of uncertain relevance to the primary care setting [24]. There is also evidence that many primary care physicians are not trained well enough to interpret statistical information on screening and favoured it despite a lack of clear evidence [25]. These results were in line with something like a “dogma of prevention”, which we observed in some of the interviewed GPs, suggesting that screening and prevention is always something valuable.

Some of the interviewed GPs partially explained the performance of unnecessary procedures with demanding patients. In a study with oncological patients, there are clues that GPs overrate patient expectations as a driver for medical overuse [26]. Further research in other patient populations is needed. Fenton et al. showed in a representative study that higher patient satisfaction not only reduces emergency department use as a positive consequence, but also correlates with “greater inpatient use, higher overall health care and prescription drug expenditures, and increased mortality” [27].

In a recent review, Chiolero et al. [28] summarised some major strategies for preventing medical overuse. However, there was no special focus on primary care or strategies that could be directly applied by GPs. The authors commonly called for a greater general awareness of medical overuse, a better training in assessing potential benefits and risks of medical procedures and watchful waiting strategies in situations with a high risk of over investigation and overtreatment.

### 3. Strengths and limitations of the study

Although medical overuse has a growing impact on health care research and policy, there has been little research from a primary care perspective so far. In this context, the strength of our study lay in its focus on the GPs’ perspective on medical overuse. We particularly aimed at exploring a broad scope and set of drivers in terms of unnecessary investigations and treatment and also strategies to reduce it (=quaternary prevention). As this focus required a qualitative study design, it was not possible to link each context of medical overuse with its individual drivers and strategies. This question will be addressed in a subsequent quantitative study.

With regard to the principle of reflexivity [29], the authors started with the preliminary hypothesis that over investigation and overtreatment represent a serious problem in primary health care which ought to be reduced through quaternary prevention strategies. However, we particularly payed attention to negative cases (GPs who thought overuse to be of no or only minor importance) in order to deal with this potential research bias [18, 29]. Throughout the iterative research process, we also expanded our semi-structured interview guide with cross-checks for rivalling explanations [29].

We did not interview secondary care specialists, patients or other stakeholders, which could have added to internal validity [29]. We suggest further research to continue with building a comprehensive theory on the phenomenon of medical overuse, taking vulnerable groups, specific diagnostic and treatment processes into account. Due to the qualitative research design and theoretical sampling of a limited number of participating GPs, the study lacks representativeness and external validity [29] in terms of a quantitative research approach.

## Conclusions

The findings of our study indicate that there are various aspects of medical overuse that can be addressed and improved in primary care itself. GPs are often located at the very starting point of a patient’s diagnostic and treatment process. They can decide whether to trigger the cascade [16] of unnecessary medicine or not. If this avalanche is already set off, it will be far more difficult to stop it than at an earlier stage. In this context, a trustful doctor-patient-relationship, patient education and the promotion of a primary care-centred health care model could be helpful. High-quality training could empower GPs to cope with residual uncertainty and to accept well-founded “wait and see”.

Research shows that GPs are open to feedback on their way of practicing [22]. This openness could be the basis of applying quaternary prevention strategies in primary care. Finally, the definition of low-value health care requires precise definitions and decisions about cut-off points regarding a net benefit or a benefit-cost ratio. This requires a careful consideration of various complex underlying normative and social implications [30]. In this context, a process of setting priorities in health care could be helpful [8].

## Additional files

Additional file 1: Interview guide – Medical overuse and quaternary prevention in primary care. (PDF 286 kb)

Additional file 2: Timeline/Mapping – Evidence-based medicine and guidelines. (PDF 1486 kb)

## Abbreviations

GP: General practitioner

## Footnotes

1 The remainder consists of special programs (for soldiers, for example) and of persons without a health insurance.
