# Supplementary material for: Medical overuse and quaternary prevention in primary care – A qualitative study with general practitioners
Source: BMC Fam Pract. 2017 Dec 8;18:99. doi: 10.1186/s12875-017-0667-4 (PMC5721694; doi:10.1186/s12875-017-0667-4)
Supplement: Supplementary file 1 — Interview guide – Medical overuse and quaternary prevention in primary care. (PDF 286 kb) [file 12875_2017_667_MOESM1_ESM.pdf]

Medical overuse and quaternary prevention in primary care – A qualitative study with general practitioners.
(Semi-structured guide for in-depth expert interviews)

A. Preliminary note

This interview guide (translated from German) was extended and adjusted with growing insight into the research topic throughout the course of the inquiry.

B. Introduction

Thank you very much for taking the time for this interview today. As we have outlined in the information sheet, our study aims at exploring and understanding medical overuse from a primary care perspective and strategies to avoid it. I am especially interested in your views and experiences as a general practitioner.

C. Questions

[Questions written in italics were only used as back-up questions/prompts, depending on the interview partner's fluency.]

1. To start with, would you tell me a bit about your professional background?

2. How would you personally define medical overuse?

3. Our study aims at exploring medical overuse from the perspective of general practitioners. Do you experience medical overuse in your daily work routine?
   a) *If YES: Question 3*
   b) *If NO: Do you experience medical overuse in other specialist groups/other general practitioners? Could you give examples? If NO: Negative case, change of interview questions, examples [Which benefit does EXAMPLE have for patients in your opinion? Are there any risks?]*

4. In which areas do you experience medical overuse?
   a) *Indications*
   b) *Special patient groups*
   c) *In-patient/out-patient care*
   d) *Discharge/referral letters*
   e) *Primary vs. secondary care*

5. Where do you experience medical overuse specifically in primary care?
   a) *Medical overuse of other general practitioners?*
   b) *In your own daily work routine?*

6. The general check-up (Check-up 35) has been controversially discussed lately. How do you feel about this examination?
   a) *In your opinion, which positive aspects do these check-ups have?*

   b) *In your opinion, which risks do these check-ups have?*
   c) *Do these check-ups have specific benefits for patients, such as increase in quality of life/decrease in morbidity or mortality?*

7. We have been talking about different areas in which you experience medical overuse. Have there been any changes in comparison to the beginning of your career?
   [Cave data analysis: Varying years of professional experience]
   a) *Are there areas in which there is more medical overuse nowadays?*
   b) *Are there areas in which there is less medical overuse nowadays?*

8. Which fields of specialisations are not prone to medical overuse?
   a) *Fields in primary care/secondary care (in-patient vs. out-patient)*
   b) *Why do you think there is no medical overuse?*

9. Do patients themselves tell you about medical overuse which they experienced?
   a) *If yes: Could you give examples?*
   b) *Which patient groups do experience medical overuse? Are there any differences?*
   c) *Are patients more/less critical towards medical overuse nowadays? Do you have an explanation for this behaviour?*

10. What are the incentives for/drivers of medical overuse in your opinion?
    a) *What do you observe in other colleagues/your daily work?*
    b) *Economic reasons, lack of experience, status of/opinions about evidence-based medicine/guidelines, skills/personality, organisational structures, defensive medicine*

11. How does a high workload influence your referral/prescription routines?
    a) *Quicker referrals, more antibiotics, no influence?*

12. General practitioners are confronted with a high level of uncertainty regarding the correct and final diagnosis. How do you feel about that?
    a) *How does diagnostic uncertainty influence your daily work routine? Could you give examples?*
    b) *Did you ever have a situation where you thought – afterwards: It would have been better not to start intensive diagnostics because it did not have any benefit for the patient (or did even harm him)?*

13. Which role do demanding patients play when unnecessary medicine is provided?
    a) *If a patient demands a medical service which is not necessary in your opinion, how will you react?*

14. We have been talking about examples and drivers of medical overuse in detail so far. How could we prevent patients and doctors from unnecessary medicine in your opinion?
    a) *In your opinion, which changes would be useful in health care?*
    b) *Doctor-patient-relationship, watchful waiting, evidence-based medicine/guidelines, re-allocation of resources*

15. Have you ever advised one of your patients NOT to pursue specific diagnostic or therapeutic regimes which were recommended by other colleagues?
    a) *How did your patient react?*
    b) *Which reasons did you give?*

16. Which stakeholders could help with preventing medical overuse?
    a) *What could general practitioners do in order to reduce medical overuse?*
    b) *What could other stakeholders in health care do to reduce medical overuse?*

17. In your opinion, which consequences does medical overuse have in the long run?

18. We are almost at the end of this interview now. Is there anything missing in your opinion? Is there anything we haven’t discussed?

19. Thank you very much for this interview and your time.
