# Supplementary material for: Medical overuse and quaternary prevention in primary care – A qualitative study with general practitioners
Source: BMC Fam Pract. 2017 Dec 8;18:99. doi: 10.1186/s12875-017-0667-4 (PMC5721694; doi:10.1186/s12875-017-0667-4)
Supplement: Supplementary file 2 — Timeline/Mapping – Evidence-based medicine and guidelines. (PDF 1486 kb) [file 12875_2017_667_MOESM2_ESM.pdf]

TIMELINE

A1 — 2014-04-08
- Guidelines/deviations
- Criticising prevention dogma

A2 — 2014-06-12
- Rationale/individual experience

A3 — 2015-01-07
- Overreliance/causal effects

A4 — 2015-01-23
- Diagnostic safeguarding/ in-patient care
- Guidelines/deviations

A5 — 2015-02-03
- Guidelines/deviations

A6 — 2015-02-04
- Guideline quality
- Guidelines/deviations

A7 — 2015-02-12
- Criticising prevention dogma
- Guideline quality
- Guidelines/deviations

A8 — 2015-03-20
- Not responsible
- Statistical knowledge
- Prevention dogma
- Little visibility/consequences
- Saving individual lives

A9 — 2015-03-20
- Guidelines/deviations

A10 — 2015-04-21
- Not responsible
- Statistical knowledge
- Prevention dogma
- Saving individual lives
- Rationale/individual experience

A11 — 2015-04-29
- Diagnostic safeguarding/ in-patient care
- Guideline quality
- Little visibility/consequences
- Guidelines/deviations

A12 — 2016-02-25
- Criticising prevention dogma
- Little visibility/consequences
- Guidelines/deviations

A13 — 2016-02-25
- Overreliance/causal effects
- Little visibility/consequences
- Rationale/individual experience

Round 1	Round 2	Round 3	Round 4
A1 to A2	A3 to A7	A8 to A11	A12 to A13

LEGEND

Guidelines/deviations
Evidence-based medicine as a way to avoid medical overuse, allowing for deviations of guidelines in individual cases

Criticising prevention dogma
GPs who criticised the belief that screening and prevention is always something good

Rationale/individual experience
Priority is on freedom of therapy, rationale for diagnostics and treatment is personal professional experience

Overreliance/causal effects
Overreliance on (causal) treatment effects achieved by applied treatment

Diagnostic safeguarding/ in-patient care
Appreciation of medical overuse in inpatient care as a welcome diagnostic work-up and baseline for the subsequent outpatient care

Guideline quality
Criticising vested interests, limited applicability to the individual patient, tendency to promote action rather than inaction

Not responsible
GPs who do not feel responsible for managing an overall evidence-based care for patients; call for health care politics, insurances, health care system instead

Statistical knowledge
Misinterpretation of statistical knowledge

Prevention dogma
Belief that screening and prevention is always something good

Little visibility/consequences
Little visibility of negative consequences of medical overuse

Saving individual lifes
"Action" dogma of doing anything that is possible for the individual patient
